# Heart Failure Probability and Early Outcomes of Critically Ill Patients With COVID-19: A Prospective, Multicenter Study

**DOI:** 10.3389/fcvm.2021.738814

**Published:** 2021-11-26

**Authors:** Weibo Gao, Jiasai Fan, Di Sun, Mengxi Yang, Wei Guo, Liyuan Tao, Jingang Zheng, Jihong Zhu, Tianbing Wang, Jingyi Ren

**Affiliations:** ^1^Department of Emergency, Peking University People's Hospital, Beijing, China; ^2^Department of Cardiology, Heart Failure Center, China-Japan Friendship Hospital, Beijing, China; ^3^Trauma Center, Peking University People's Hospital, Beijing, China; ^4^Research Center of Clinical Epidemiology, Peking University Third Hospital, Beijing, China

**Keywords:** COVID-19, heart failure, NT-ProBNP, nomogram, prognosis

## Abstract

**Background:** The relationship between cardiac functions and the fatal outcome of coronavirus disease 2019 (COVID-19) is still largely underestimated. We aim to explore the role of heart failure (HF) and NT-proBNP in the prognosis of critically ill patients with COVID-19 and construct an easy-to-use predictive model using machine learning.

**Methods:** In this multicenter and prospective study, a total of 1,050 patients with clinical suspicion of COVID-19 were consecutively screened. Finally, 402 laboratory-confirmed critically ill patients with COVID-19 were enrolled. A “triple cut-point” strategy of NT-proBNP was applied to assess the probability of HF. The primary outcome was 30-day all-cause in-hospital death. Prognostic risk factors were analyzed using the least absolute shrinkage and selection operator (LASSO) and multivariate logistic regression, further formulating a nomogram to predict mortality.

**Results:** Within a 30-day follow-up, 27.4% of the 402 patients died. The mortality rate of patients with HF likely was significantly higher than that of the patient with gray zone and HF unlikely (40.8% vs. 25 and 16.5%, respectively, *P* < 0.001). HF likely [Odds ratio (OR) 1.97, 95% CI 1.13–3.42], age (OR 1.04, 95% CI 1.02–1.06), lymphocyte (OR 0.36, 95% CI 0.19–0.68), albumin (OR 0.92, 95% CI 0.87–0.96), and total bilirubin (OR 1.02, 95% CI 1–1.04) were independently associated with the prognosis of critically ill patients with COVID-19. Moreover, a nomogram was developed by bootstrap validation, and C-index was 0.8 (95% CI 0.74–0.86).

**Conclusions:** This study established a novel nomogram to predict the 30-day all-cause mortality of critically ill patients with COVID-19, highlighting the predominant role of the “triple cut-point” strategy of NT-proBNP, which could assist in risk stratification and improve clinical sequelae.

**Graphical Abstract:**
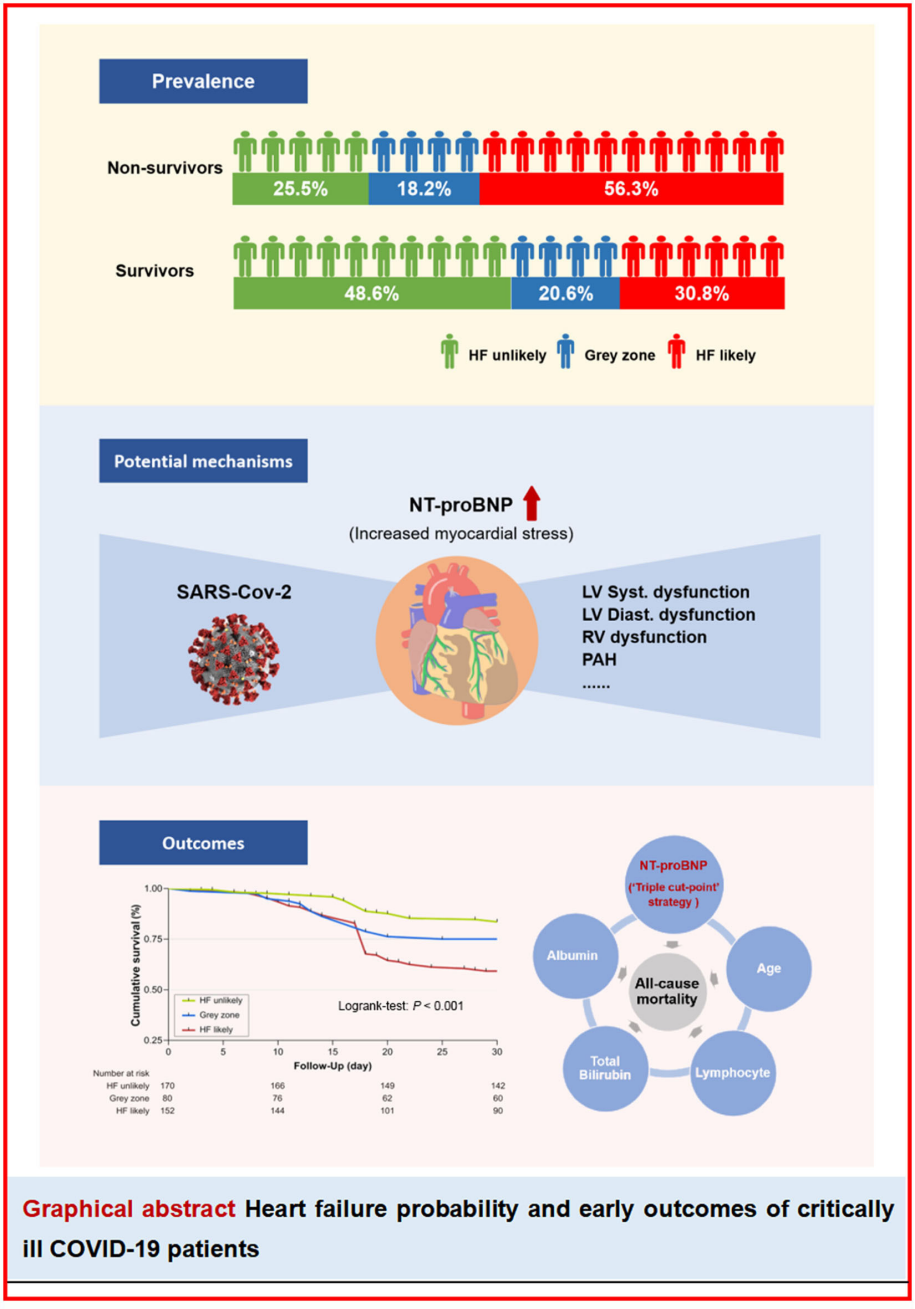
Heart failure probability and early outcomes of critically ill COVID-19 patients. HF, heart failure; NT-proBNP, N-terminal probrain natriuretic peptide; LV, left ventricular; RV, right ventricular; PAH, pulmonary arterial hypertension.

## Introduction

An outbreak of novel infectious pneumonia, now known as coronavirus disease 2019 (COVID-19) caused by severe acute respiratory syndrome coronavirus 2, has been quickly spreading around the world since December 2019. To date, more than 168 million confirmed cases of COVID-19 have been identified worldwide, with over 3.49 million deaths. Despite the advancement of learning the etiology and clinical characteristics of COVID-19, there have been no effective strategies to wipe out the global COVID-19 epidemic, and it is still a public health threat.

The average mortality rate was estimated globally at 3.4% by the WHO, while it is 26–52% significantly higher for patients admitted to intensive care units (ICUs) (1, 2). Moreover, no medications have been proven definitely effective for curing COVID-19 (3). Thus, early evaluation and identification of individuals with high-risk mortality are of paramount importance to further guide optimal intervention strategies. Of note, critically ill patients with COVID-19 usually have multiple organ dysfunctions (4), among which cardiac involvement is prevalent, especially acute heart failure (AHF) (5). However, the role of AHF in the prognosis of COVID-19 has not been fully elucidated in prior studies, partially because comprehensive evaluations of cardiac dysfunction that utilize imaging examinations were usually unavailable in real-world practice. Hence, we proposed a “triple cut-point” strategy of N-terminal pro-brain natriuretic peptide (NT-proBNP) as a reliable and easy-to-use diagnostic tool for AHF in this study (6). Moreover, although previous studies have explored the risk factors of prognosis among critically ill patients with COVID-19 (7–9), a user-friendly and clinically relevant short-time outcome prediction model for patients with COVID-19 in ICU is still lacking.

Therefore, to address the gaps mentioned above, this multicenter study aims to (1) explore the potential prognostic value of the “triple cut-point” strategy of NT-proBNP and AHF in critically ill patients with COVID-19; and (2) construct and validate a simplified and effective nomogram to predict all-cause in-hospital death risk individually.

## Materials and Methods

### Study Population

This multicenter, prospective, and observational study consecutively enrolled 1,050 patients with clinical suspicion of COVID-19 from four ICUs in Wuhan taken over by China-Japan Friendship Hospital, Peking University People's Hospital, Peking University First Hospital, and Peking University Third Hospital from January to May 2020. Patients who met one of the following criteria would be considered to be transferred to the ICU: (1) respiratory rate >30 breath/min; (2) blood oxygen (SpO_2_) <93%; (3) PaO_2_/FiO_2_ <300 mmHg; (4) presented with respiratory failure; (5) presented with shock; or (6) other conditions that need to be monitored in the ICU. Patients were diagnosed as COVID-19 with a positive result of real-time reverse transcriptase-polymerase chain reaction assay from nasal swab specimens according to WHO guidance (10). Exclusion criteria included patients who were not diagnosed with COVID-19, younger than 18 years of age, and had incomplete data, or died within 24 h of admission to the ICU. As a result, 402 patients were included in the final analysis. The study was conducted in accordance with the Declaration of Helsinki, and the protocol was approved by the Ethics Committee of Peking University People's Hospital.

### Data Collection

The clinical data from each patient were recorded by experienced physicians following ICU admission and included demographic features, preexisting comorbidities, symptoms, vital signs, and length of ICU stay. The comorbidities included hypertension, coronary heart disease (CHD), diabetes mellitus (DM), chronic kidney disease (CKD), asthma, chronic obstructive pulmonary disease, chronic bronchitis, transient ischemic attack, ischemic stroke, and hemorrhagic stroke.

All the patients, during hospitalization, were followed up for 30-days or until discharge or death. The primary outcome was 30-day all-cause death after admission.

### Laboratory Measurements

Laboratory values were collected including complete blood count, high-sensitivity cardiac troponin I (hs-cTNI), NT-proBNP, biochemical tests, d-dimer, and procalcitonin (PCT). Complete blood count was measured with a Sysmex XN-9000 (Sysmex, Kobe, Japan) automatic hematology analyzer. Coagulation parameters, such as d-dimer, were measured with a Stago STA-R automatic blood coagulation analyzer (Stago, Paris, France). Biochemical tests, namely, alanine aminotransferase (ALT), aspartate aminotransferase (AST), total bilirubin, albumin, blood urea nitrogen (BUN), and creatinine were performed using Roche Cobas 8000 automatic biochemical analyzer (Roche, Rotkreuz, Switzerland). Hs-cTNI was measured with an Abbott ARCHITECT i2000SR chemiluminescence immunoanalyzer (Abbott Laboratories, Illinois, United States). Elevated hs-cTNI was defined as plasma levels of hs-cTNI above the 99th-percentile upper reference limit. NT-proBNP was analyzed with a Roche Cobas e602 electrochemical luminescence analyzer (Roche, Germany).

### “Triple Cut-Point” Strategy of NT-proBNP

According to the recent guideline for HF, novel NT-proBNP cut-off values have been proposed to assist with AHF diagnosis. Hence, we classified the cases into three groups using this “triple cut-point” strategy of NT-proBNP to define the probability of AHF, as shown in Table 1. In detail, HF likely was defined as plasma NT-proBNP level > 450 pg/ml in patients below 50 years, >900 pg/ml in patients between 50 and 75 years, and >1,800 pg/ml in patients over 75 years (6). HF unlikely was defined as plasma NT-proBNP level <300 pg/ml regardless of age, while the stratified approach of 300 pg/ml to 450/900/1,800 pg/ml for ages <50/50–75/>75 years were considered as “gray zone.”

**Table 1:** Classification of patients using the “triple cut-point” strategy of NT-proBNP.

**Setting**	**Cut-off levels of NT-proBNP (pg/mL)**		
	**Age < 50**	**Age 50–75**	**Age > 75**
HF unlikely	<300		
Gray zone	300–450	300–900	300–1,800
HF likely	>450	>900	>1,800

*NT-proBNP, N-terminal pro-B type natriuretic peptide; HF, heart failure*.

### Statistical Analysis

Continuous variables were presented as mean ± SD if normally distributed, and median and interquartile range otherwise. The differences between the two groups were compared by the Student *t*-test and Mann–Whitney U test appropriately. Categorical variables were shown as n (%) and compared by χ^2^ test or Fisher exact test when necessary.

Kaplan–Meier survival estimates were calculated, and the log-rank test was performed to compare the groups in terms of survival. The least absolute shrinkage and selection operator (LASSO) method (glmnet package), which is appropriate for regression of high-dimensional data, was used to select the most useful predictive variables from the data set. Then, the multivariate logistic regression analysis was performed to identify independent risk factors. Odds ratios (ORs) were shown with a 95% CI.

The nomogram was established based on the multivariate logistic regression analysis (rms package). A likelihood ratio test approach for model selection was performed. Nomogram performance was quantified with respect to discrimination and calibration. Discrimination (the ability of a nomogram to separate patients with all-cause in-hospital death) was quantified with the concordance index (C-index) and 95% CI. Calibration was assessed graphically by plotting the relationship between actual (observed) probabilities and predicted probabilities (calibration plot) by Hosmer goodness-of-fit test. The internal validation of performance was estimated with the bootstrapping method (500 replications). Integrated discrimination improvement (IDI) and net reclassification improvement (NRI) (survival package) were used to assess the improved ability of the “triple cut-point” strategy of NT-proBNP for the predictive value of the model.

All the tests were two-tailed, and a *P* < 0.05 was considered significant. The statistical analyses were performed with the SPSS version 25.0 software (SPSS Inc., Chicago, IL, United States), R programming language, and environment version 3.6.0 (http://cran.r-project.org).

## Results

### Baseline Characteristics

This study finally included 402 critically ill patients with laboratory-confirmed COVID-19 and their baseline characteristics are shown in Table 2. Overall, the mean age of the whole cohort was 67 years, and 54.5% (*n* = 219) were men. At the 30-day follow-up, 110 patients had died with a 27.4% mortality risk. Compared to the survivors, the non-survivors were more likely to be older, having decreased SpO_2_ and elevated heart rate (HR) (all *P* < 0.05). Of note, there was no significance between the two groups regarding gender and comorbidities, irrespective of hypertension, CHD, DM, and respiratory system disease. Furthermore, we compared the laboratory data between the two groups and found that the non-survivors had significantly increased AST, total bilirubin, blood glucose, d-dimer, and PCT as well as decreased lymphocytes, platelets, and estimated glomerular filtration rate (eGFR) (all *P* < 0.05).

**Table 2:** Baseline characteristics of the cohort.

**Demographics**	**Total** ***n*** **= 402 (100.0)**	**Survivors** ***n*** **= 292 (72.6)**	**Non-survivors** ***n*** **= 110 (27.4)**	***P*** **-value**
Age, years	67.5 ± 13.7	65.6 ± 13.6	72.4 ± 12.8	<0.001
Sex				0.070
Female, *n* (%)	183 (45.5)	141 (48.3)	42 (38.2)	
Male, *n* (%)	219 (54.5)	151 (51.7)	68 (61.8)	
**Vital signs**				
Temperature, °C	38.7 ± 3.7	38.6 ± 4.3	39.0 ± 1.0	0.332
Respiratory rate, breath/min	25.1 ± 6.1	25.0 ± 5.6	25.4 ± 7.2	0.603
SpO_2_, %	91.2 ± 6.7	92.2 ± 5.7	88.6 ± 8.3	<0.001
Heart rate, beat/min	94.7 ± 17.0	93.7 ± 15.6	97.3 ± 20.0	0.002
SBP, mm/Hg	133.0 ± 23.0	132.6 ± 22.4	134.0 ± 24.5	0.570
DBP, mm/Hg	79.0 ± 14.4	78.9 ± 14.0	79.2 ± 15.3	0.883
**Comorbidities**				
Hypertension, *n* (%)	209 (52.0)	152 (52.1)	57 (51.8)	0.966
Coronary heart disease, *n* (%)	72 (17.9)	48 (16.4)	24 (21.8)	0.210
Diabetes mellitus, *n* (%)	96 (23.9)	69 (23.6)	27 (24.5)	0.848
Respiratory system diseases, *n* (%)	51 (12.7)	37 (12.7)	14 (12.7)	0.988
Chronic kidney disease, *n* (%)	37 (9.2)	25 (8.6)	12 (10.9)	0.468
Cerebrovascular diseases, *n* (%)	19 (4.7)	11 (3.8)	8 (7.3)	0.140
Cardiac comorbidities or risk factors, *n* (%)	258 (64.2)	187 (64.0)	71 (64.5)	0.925
No. of comorbidities ≥2, *n* (%)	139 (34.6)	95 (32.5)	44 (40.0)	0.161
**Laboratory values**				
Lymphocyte, × 10^9^ /L	0.8 (0.5, 1.1)	0.8 (0.7, 1.2)	0.6 (0.4, 0.8)	<0.001
Platelet, × 10^9^ /L	217.0 (141.8, 289.0)	235.0 (157.0, 307.2)	160 (106.0, 225.2)	<0.001
ALT, U/L	30.0 (16.4, 47.0)	28.5 (16.0, 46.0)	33.1 (18.0, 48.3)	0.291
AST, U/L	34.0 (21.0, 52.8)	29.2 (20.0, 50.8)	43.5 (26.4, 57.0)	<0.001
Albumin, g/L	32.2 ± 5.9	33.0 ± 5.2	29.9 ± 6.9	<0.001
Total bilirubin, μmol/L	12.2 (8.6, 16.9)	11.4 (8.3, 15.3)	14.5 (9.7, 20.4)	<0.001
eGFR, ml/ min/l.73 m^2^	73.2 ± 31.7	76.0 ± 31.8	65.7 ± 30.2	0.003
Glucose, mmol/L	8.6 ± 4.1	8.3 ± 4.0	9.5 ± 5.8	0.021
D-dimer, μg/mL	2.5 (0.7, 20.1)	1.7 (0.5, 15.7)	7.3 (2.4, 20.1)	<0.001
PCT, ug/L	0.3 (0.1, 1.8)	0.2 (0.1, 1.2)	1.5 (0.3, 1.8)	<0.001
hs-cTNI, pg/mL	12.0 (2.4, 503.7)	10.2 (2.3, 80.1)	274.4 (6.0, 659.6)	<0.001
NT-proBNP, pg/mL	393.2 (121.5, 2774.8)	321.0 (105.0, 2774.8)	1563.5 (240.7, 2775.8)	<0.001

*Data are expressed as mean ± SD, median (25th−75th percentile), or n (%). SpO_2_, oxygen saturation; SBP, systolic blood pressure; DBP, diastolic blood pressure; ALT, alanine aminotransferase; AST, aspartate aminotransferase; eGFR, estimated glomerular filtration rate; PCT, procalcitonin; hs-cTNI, higher sensitivity cardiac troponin I; NT-proBNP, N-terminal pro-brain natriuretic peptide*.

### “Triple Cut-Point” Strategy of NT-proBNP and 30-Day Mortality

**Figure 1:**
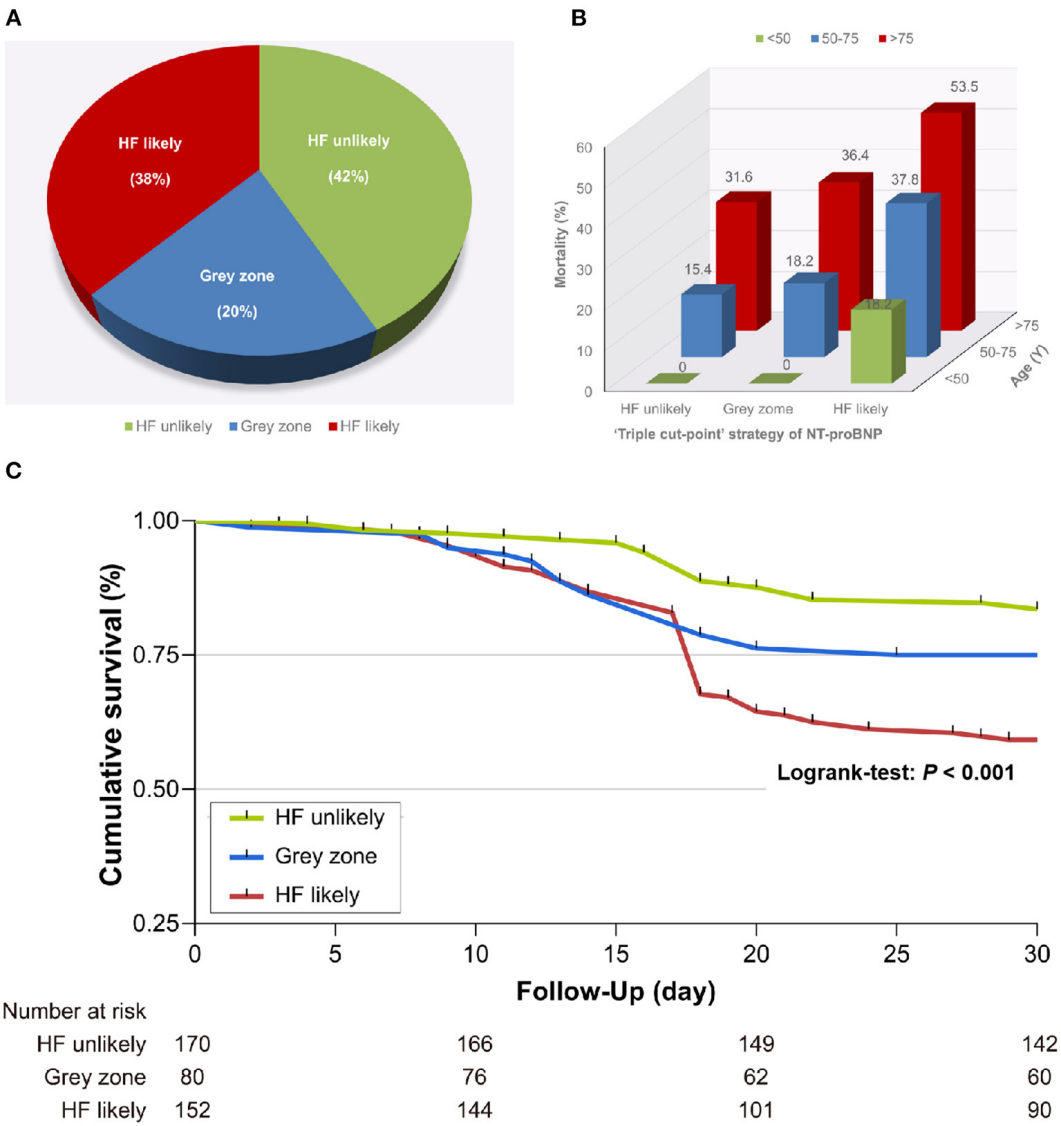
Relationship between the “triple cut-point” strategy of N-terminal pro-brain natriuretic peptide (NT-proBNP) and death. **(A)** Distribution of the “triple cut-point” strategy of NT-proBNP (*n* = 402). **(B)** The mortality rate increased with aging and heart failure. **(C)** Kaplan–Meier survival curves stratified by the “triple cut-point” strategy of NT-proBNP. HF, heart failure.

According to the “triple cut-point” strategy of NT-proBNP, the patients were divided into three groups, and the overall distribution of HF unlikely, gray zone, and HF likely was 170 (42.3%), 80 (19.9%), and 152 (37.8%), respectively (Figure 1A). As shown in Table 3, the non-survivor group has a significantly higher percentage of patients with HF likely (56.3 vs. 30.8%), and the distribution of the three groups (HF unlikely, gray zone, and HF likely) between the non-survivor and survivor groups was significantly different (*P* < 0.001). Otherwise, within the 30-day follow-up, we observed a mortality rate of 16.5 (28/170), 25 (20/80), and 40.8% (62/152) in group HF unlikely, gray zone, and HF likely, respectively (*P* < 0.001). Importantly, the mortality rate increased sharply, accompanied by the increased likelihood of AHF (Figure 1B). The Kaplan-Meier curves of short-time survival were shown in the central illustration, illustrating a significantly shorter mean survival time for patients with HF likely (Figure 1C). The overall cumulative risk of death at 30-days was significantly higher for the HF likely group than for HF unlikely and gray zone (*P* < 0.001).

**Table 3:** Distribution of “triple cut-point” strategy of NT-proBNP in critically ill patients with coronavirus disease 2019 (COVID-19).

**Setting**	**Total** ***n*** **= 402 (100.0)**	**Survivors** ***n*** **= 292 (72.6)**	**Non-survivors** ***n*** **= 110 (27.4)**	***P*** **-value**
HF unlikely, *n* (%)	170 (42.3)	142 (48.6)	28 (25.5)	<0.001
Gray zone, *n* (%)	80 (19.9)	60 (20.6)	20 (18.2)	
HF likely, *n* (%)	152 (37.8)	90 (30.8)	62 (56.3)^*^	

*Data are expressed as n (%). NT-proBNP, N-terminal pro-brain natriuretic peptide; HF, heart failure.*

* *The distribution of HF likely between survivors and non-survivors was confirmed to be significantly different by post-hoc test (p < 0.001)*.

### Predictors of 30-Day in-hospital Death of Critically Ill Patients With COVID-19

**Figure 2:**
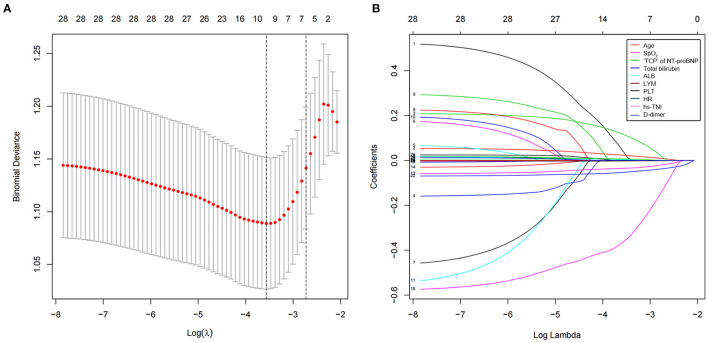
Clinical feature selection using a least absolute shrinkage and selection operator (LASSO) binary logistic regression model. **(A)** Selection of optimal parameters (lambda) from the LASSO model using 10-fold cross-validation and minimum criteria. The partial likelihood deviance (binomial deviance) curve was plotted vs. log (lambda). Dotted vertical lines were drawn at the optimal values using the minimum criteria and the 1 standard error of the minimum criteria (1-SE criteria). **(B)** LASSO coefficient profiles of the 28 texture features. A vertical line was drawn at the value selected using 10-fold cross-validation, where optimal l resulted in nine non-zero coefficients. LASSO, least absolute shrinkage and selection operator; hs-cTNI, higher sensitivity cardiac troponin I; “TCP” of NT-proBNP, “triple cut-point” strategy of NT-proBNP; SpO_2_, blood oxygen; HR, heart rate.

The least absolute shrinkage and selection operator was used to select the potential prognostic factors from numerous parameters. Finally, 28 indexes were reduced to 10 potential predictors, namely, age, lymphocyte, platelet, total bilirubin, "triple cut-point” strategy of NT-proBNP, SpO_2_, HR, albumin, hs-cTNI, and d-dimer, based on the 402 patients, and were indexes with non-zero coefficients in the LASSO regression model (Figures 2A,B). Furthermore, as shown in Table 4, the multivariate logistic regression analysis displays five independent predictors for the short-time fatal outcome, namely, HF likely (OR 1.97, 95% CI 1.133–3.424), older age (OR 1.04, 95% CI 1.018–1.061), lymphocyte (OR 0.361, 95% CI 0.191–0.681), total bilirubin (OR 1.022, 95% CI 1.002–1.042), and albumin (OR 0.915, 95% CI 0.87–0.963) (all *P* < 0.05).

**Table 4:** Multivariate logistic regression analyses of risk factors for 30-day mortality.

**Variable**	Univariate analysis^*^		**Multivariate analysis**	
	**OR (CI 95%)**	***P-*** **value**	**OR (CI 95%)**	***P-*** **value**
Age, years	1.042 (1.022, 1.061)	<0.001	1.040 (1.018, 1.061)	<0.001
Spo_2_, %	0.926 (0.894, 0.959)	<0.001	—	
HR, beat/min	1.012 (1.000, 1.025)	0.060	—	
PLT, × 10 ^9^ /L	0.994 (0.992, 0.996)	<0.001	—	
LYM, × 10 ^9^ /L	0.224 (0.120, 0.418)	<0.001	0.361 (0.191, 0.681)	0.002
Albumin, g/L	0.878 (0.837, 0.921)	<0.001	0.915 (0.870, 0.963)	0.001
Total bilirubin, μmol/L	1.032 (1.010, 1.054)	0.005	1.022 (1.002, 1.042)	0.032
hs-cTNI, pg/mL	1.010 (1.001, 1.023)	0.048	—	
“TCP” of NT-proBNP		<0.001		0.013
HF unlikely	Reference	—	Reference	—
Gray zone	1.367 (0.718, 2.584)	0.343	1.011 (0.425, 1.725)	0.665
HF likely	2.773 (1.678, 4.583)	<0.001	1.970 (1.133, 3.424)	0.016
D-dimer, μg/mL	1.053 (1.027, 1.080)	<0.001	—	

* *The variables of the univariate analysis were from the least absolute shrinkage and selection operator (LASSO) binary logistic regression model.*

*Spo_2_, blood oxygen; HR, heart rate; hs-cTNI, higher sensitivity cardiac troponin I; “TCP” of NT-proBNP, “triple cut-point” strategy of NT-proBNP*.

### Development and Validation of a Novel Nomogram for Predicting Prognosis

**Figure 3:**
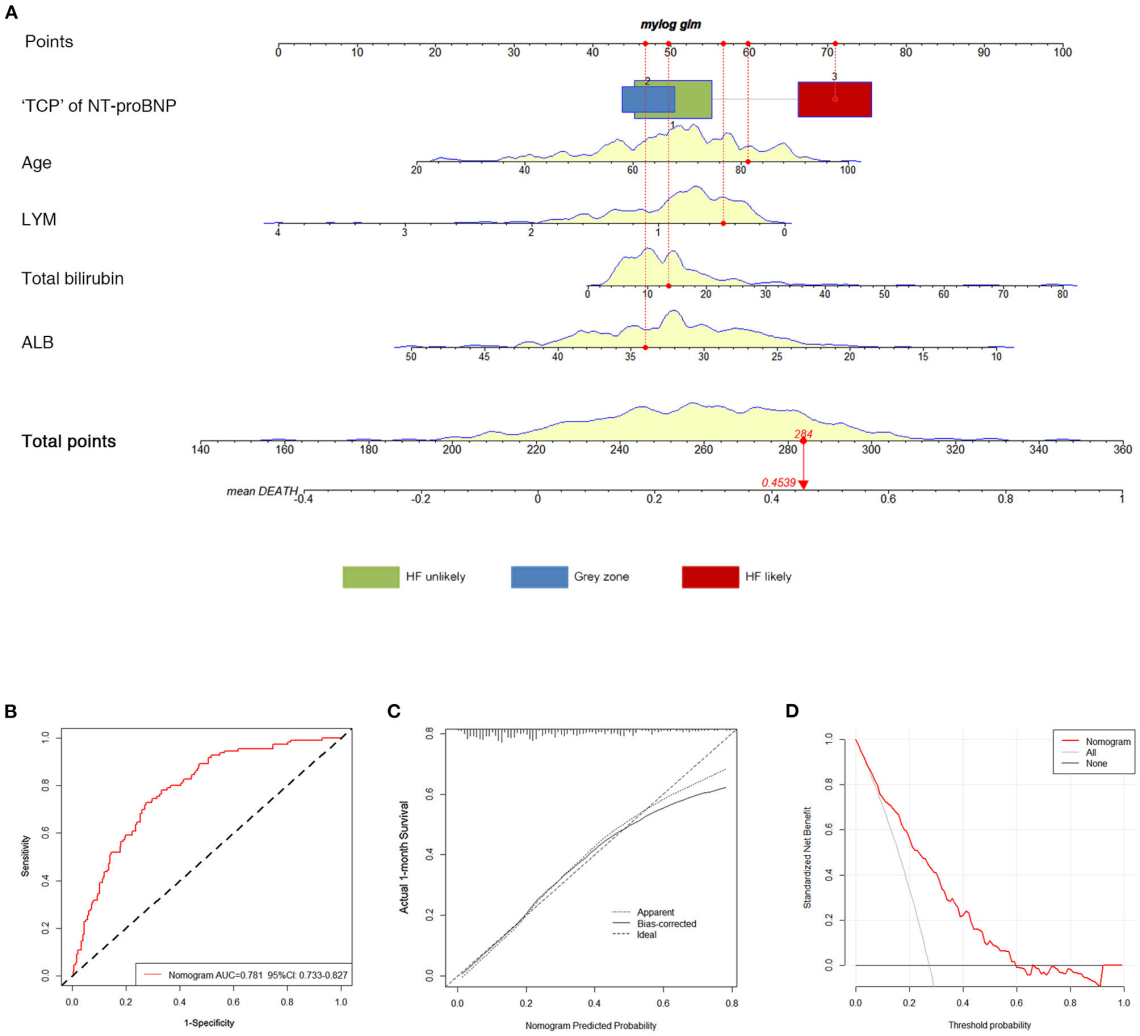
Construction and validation of the nomogram for 30-day all-cause death. **(A)** The total nomogram point of each patient can be used to predict death risk on an individual basis. To predict patient death risk at 30-days, take the following as an example: an 81-year-old patient (60 points) who belonged to HF likely (71 points) had albumin of 34 g/L (46.5 points); his lymphocyte was.5 × 10^9^ /L (56.5 points), and total bilirubin was 14 μmol/L (50 points) at admission. He has a total point score of 284, corresponding to a 45.39% risk of death at 30-days. **(B)** ROC curves of the nomogram. **(C)** Calibration plot of observed proportion vs. predicted probability of 30-day death of the nomogram. **(D)** DCA for the nomogram and the model with subtracting of HF. The y-axis measures the net benefit. The dotted pink line represents the nomogram. The thin gray line represents the assumption that all patients will die. The bold black line represents the assumption that no patient will die. “TCP” of NT-proBNP, “triple cut-point” strategy of NT-proBNP; HF, heart failure; AUC, the area under the curve; ROC, receiver operating characteristic; DCA, decision curve analysis.

An optimal nomogram comprising all the above independent predictors was established to individualize the risk of 30-day in-hospital death (Figure 3A). The ratios of calculated β were used to decide the proportional prognostic effect of these variables. Projections from total points on the scales below indicated the estimated probability of death.

Performance accuracy was evaluated by the area under the curve (AUC) of the receiver operating characteristic (ROC) analysis. The AUC for in-hospital death was 0.781 (95% CI 0.733–0.827) (Figure 3B). The calibration curve of the nomogram for the probability of death demonstrated good agreement between prediction and observation in the primary cohort (Figure 3C). Hosmer-Lemeshow goodness-of-fit was satisfied (*P* = 0.354). The C-index for the prediction nomogram was 0.798 (95% CI 0.742–0.857). The decision curve analysis (DCA) for the clinical laboratory index nomogram is presented in Figure 3D. It showed that this nomogram had more benefits than the treat-all-patients scheme or the treat-none scheme in predicting the risk of 30-day in-hospital death of critically ill patients with COVID-19. Moreover, the bootstrap validation method was used to verify the predictive accuracy of the nomogram. The C-index for the nomogram of 30-day in-hospital death was 0.779 (95% CI 0.721–0.834), suggesting the accuracy of this predictive nomogram.

### Incremental Predictive Value of “Triple Cut-Point” Strategy of NT-proBNP

To investigate the role of the “triple cut-point” strategy of NT-proBNP in the predictive value of the current model, NRI and IDI were calculated. Compared with the model without the “triple cut-point” strategy of NT-proBNP, the addition of the “triple cut-point” strategy of NT-proBNP resulted in a significantly improved discrimination [IDI 7.3% (95% CI 1.1–14.5%) and NRI 4.9% (95% CI 2.6–7.2%), both with *P* < 0.05].

## Discussion

In this prospective multicenter study, we recruited 402 critically ill patients with COVID-19 from four ICUs in China and established a novel nomogram to predict the 30-day all-cause mortality risk in these patients. To the best of our knowledge, there have been few risks score models for predicting the prognosis of critically ill patients with COVID-19. This study has developed a user-friendly and relatively personalized model incorporating five variables, age, “triple cut-point” strategy of NT-proBNP, albumin, lymphocyte count, and total bilirubin, to predict short-time mortality risk in critically ill Chinese patients with COVID-19, which could assist risk stratification and provide insights for timely interventions upon admission. Furthermore, it is highlighted that the “triple cut-point” strategy of NT-proBNP demonstrated the predominant role of AHF in the clinical course and prognosis in COVID-19.

The mortality risk of COVID-19 has been proven high, with 28-day mortality ranging from 26–53.8% in critically ill adult patients worldwide (1, 11), indicating the imperative of proposing an easy-to-use prediction model to assist risk-stratify and therapeutic optimization in clinical practice for ICU patients. Emerging evidence has tried to explore the risk factors and construct diagnostic and prognostic models in COVID-19 populations (12, 13). However, previous reports regarding prognosis prediction have mainly focused on disease progression or mortality risk of the whole group without further distinguishing critically ill patients in ICU (14). The sample selection bias of the prior models could lead to poor adaptions. In line with existing data, this study reported a 27.6% mortality risk, and further constructed and validated a novel nomogram for the prediction of 30-day all-cause death. Variables referring to older age, higher level of total bilirubin, lower level of lymphocyte count and albumin, and “triple cut-point” strategy of NT-proBNP were likely to recognize individuals who are at high risk with high sensitivity and specificity. More importantly, the quantitative appraisal made it possible to estimate the likelihood of death more accurately and individually with easy and rapid access in clinical practice.

It has been widely confirmed that cardiac involvement, such as cardiac injury, arrhythmias, myocarditis, and cardiac dysfunction, was prevalent and prognostic in hospitalized patients with COVID-19 (15–17), among which HF is responsible for substantial morbidity and mortality (10). New-onset HF was observed in nearly 23% of hospitalized patients with COVID-19 and as much as one-third of those admitted to the ICU (2, 9). Recent reports revealed that HF was the most frequent cause of death just after acute respiratory distress syndrome (ARDS) and sepsis, accounting for 27.4% of the proximate causes of death in patients with COVID-19 (18). However, the role of HF in the prognosis of critically ill patients with COVID-19 has not been fully elucidated, partially because of high diagnostic uncertainty. A complete diagnosis of HF usually includes symptoms, signs, biomarkers (BNP/NT-proBNP), and imaging examinations, while it is impractical and unavailable to evaluate cardiac function by echocardiography for each critically ill patient with COVID-19 in the clinical practice. Although BNP/NT-proBNP levels are easily interfered with and obscured by considerable factors, the utility of these biomarkers performed well in the emergency setting as an adjunct tool for the diagnosis and triage of dyspneic patients. As such, the guidance has recommended BNP/NT-proBNP as a diagnostic aid for HF with comparable diagnostic accuracy.

As the widely admitted biomarker in HF, NT-proBNP quantitatively reflects hemodynamic myocardial stress (19), indicating not only left ventricular (LV) systolic dysfunction but also cardiac abnormalities, such as LV diastolic dysfunction, right ventricular (RV) dysfunction, valvular dysfunction, increased pulmonary pressures, and atrial arrhythmias. Prior studies have observed ambiguous results that higher levels of BNP or NT-proBNP were found in patients with severe COVID-19 and that they were independently associated with high mortality, maybe because of single-center design, patient population selection bias, and small sample size (20–23). This multicenter study demonstrated that the non-survivors had a significantly higher level of NT-proBNP than the survivors (1,564 vs. 321 ng/ml), with reasonable sample size. Consistently, a recent study described the characterization of NT-proBNP in patients with COVID-19, and 48.5% of their cohort presented NT-proBNP levels above the recommended cut-off for the identification of HF (24).

Furthermore, considering the fact that the plasma level of NT-proBNP is largely affected by age and renal functions, it seems to be not rigorous enough to use NT-proBNP as a simple continuous variable alone to predict the prognosis of patients with COVID-19. Thus, we reclassified the subjects into three groups (HF likely, gray zone, and HF unlikely) according to the recent HF guidance as to the “triple cut-point” strategy of NT-proBNP, and observed that patients with HF likely occupied 37.8% of the total cohort, of which 56.3% were non-survivors (6). Moreover, we found that patients in the HF likely group had a significantly higher risk (OR 1.97, 95% CI 1.133–3.424) for 30-day all-cause death. Concerning the clinical presentations and biomarkers of HF on time would help make optimal individual treatment plans to prevent further deterioration efficiently.

It is worth noting that our prediction model did not incorporate troponin, as it was not independently associated with the outcome unexpectedly, while prior studies have suggested that troponin was a significant prognostic indicator in COVID-19 (15, 25). Similarly, Dong et al. conducted a retrospective study and built a nomogram assessing the 14-day and 21-day in-hospital survival of all the general patients with COVID-19. The final model was constructed based on hypertension, neutrophil-to-lymphocyte ratio, and NT-proBNP (26). Elevated troponin may be a possible confounder for NT-proBNP as they were postulated to share the same pathophysiological processes and found to be both elevated in pneumonia, sepsis, ARDS, and several other non-cardiac illnesses. Hence, we speculated that an accurate classification using the “triple cut-point” strategy of NT-proBNP may decrease the confounding effect of troponin. Notably, liver injuries, such as elevated total bilirubin and decreased albumin, have also been demonstrated to be common and associated with disease severity and poor outcomes for critically ill patients in this study, in accordance with previous studies (27). Furthermore, elevated total bilirubin may also be associated with cardiac dysfunctions as a significant and independent predictor of poor cardiovascular prognosis in patients with HF (28).

Critically ill conditions with COVID-19 were usually complicated by multiple organ dysfunctions with complex pathophysiological processes involving numerous parameters, including but not limited to hypoxemia, inflammation, thromboembolism, renal failure, and cardiac damage (29). Therefore, it is of vital importance to bring all reasonable possible variables into analysis and construct a scientific prediction model relying on appropriate statistical analysis awfully. In the current study, LASSO, a machine learning algorithm, was applied to shrink the regression coefficients from amounting clinical and laboratory indicators to 10 potential predictors. Thus, this algorithm could conquer common confusing collinearity issues and yield more robust results than traditional variable screening methods.

Some cautions should be considered when interpreting our results. First, although our study is observational and the sample size is relatively small, it has a multicenter and prospective design emphasizing critically ill patients. Further investigations with a larger sample size are warranted. Second, this study did not apply other diagnostic tools to make a complete HF diagnosis. However, it is impractical and unavailable to evaluate cardiac functions by echocardiography for each critically ill patient with COVID-19. Conversely, rapid measurements of NT-proBNP have substantial medical aids to fulfill the clinical need underlying this extraordinary stressful setting, although it should never be a stand-alone test for HF diagnosis. Third, our nomogram model lacks validation in an external population. Nevertheless, internal verification indicated the predictive strength in our study.

## Conclusions

In this study, we explored the independent predictors for short-time prognosis in critically ill patients with COVID-19 in China and established a novel nomogram to predict the 30-day all-cause mortality risk for the first time, highlighting the predominant role of the “triple cut-point” strategy of NT-proBNP. This easy-to-use prognostication nomogram can provide survival estimations and help identify patients with COVID-19 with a high-risk trajectory, further advancing clinical management and ultimately improving outcomes.

## Data Availability Statement

The raw data supporting the conclusions of this article will be made available by the authors, without undue reservation.

## Ethics Statement

The studies involving human participants were reviewed and approved by the Ethics Committee of Peking University People's Hospital. The ethics committee waived the requirement for written informed consent.

## Author Contributions

JR and TW conceived and designed the study and coordinated to complete the study. JR critically revised the manuscript. WG contributed to data collection and completed the project. JF and DS analyzed and interpreted the data and wrote the manuscript. LT assisted in performing statistical analysis. MY, WG, JZhe, and JZhu helped revise the manuscript for important intellectual content and language polishing. All the authors have read and approved the final version of the manuscript.

## Funding

This study was supported by the National Natural Science Foundation of China (81770359 to JR), State Key Laboratory of Molecular Developmental Biology of China (2020-MDB-KF-17 to JR), Beijing Health Science and Technology Achievements and Appropriate Technology Promotion Project (BHTPP202004 to JR), Elite Medical Professionals project of China-Japan Friendship Hospital (ZRJY2021-BJ01 to JR), and China-Japan Friendship Hospital Scientific Research Funds (2019-2-QN-77 to DS).

## Conflict of Interest

The authors declare that the research was conducted in the absence of any commercial or financial relationships that could be construed as a potential conflict of interest.

## Publisher's Note

All claims expressed in this article are solely those of the authors and do not necessarily represent those of their affiliated organizations, or those of the publisher, the editors and the reviewers. Any product that may be evaluated in this article, or claim that may be made by its manufacturer, is not guaranteed or endorsed by the publisher.
